# Gastric Bypass Sensitizes Sympathetic and Thermogenic Activity of Brown Adipose Tissue to Cold Exposure

**DOI:** 10.1007/s11695-021-05560-1

**Published:** 2021-07-15

**Authors:** Yi Chu, Liping Tian, Hussein Herz, Benjamin Linden, Donald A. Morgan, Meghan C. Naber, Matthew Potthoff, Kamal Rahmouni, Mohamad Mokadem

**Affiliations:** 1 Department of Internal Medicine, University of Iowa Carver College of Medicine, Iowa City, IA 52242 USA; 2 Department of Neuroscience and Pharmacology, University of Iowa Carver College of Medicine, Iowa City, IA 52242 USA; 3 Fraternal Order of Eagles Diabetes Research Center, University of Iowa, Iowa City, IA 52242 USA; 4 Obesity Research & Education Initiative, University of Iowa, 200 Hawkins Drive, 4570, Iowa City, IA 52242 USA; 5 Iowa City Veterans Affairs Health Care System, Iowa City, IA 52242 USA

**Keywords:** Gastric bypass, Brown adipose tissue, Thermogenesis, Sympathetic nerve activity, Cold exposure

## Abstract

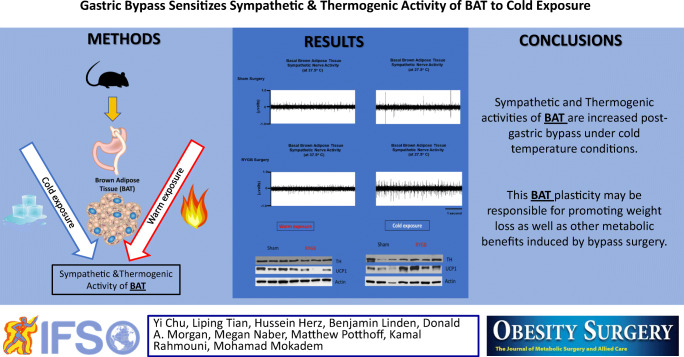

## Introduction

Multiple previous animal studies showed that Roux-en-Y gastric bypass (RYGB) induces an increase in total energy expenditure, specifically the resting metabolic rate [1–4]. It was recently demonstrated that this increase in resting metabolic rate is due to a sympathetically mediated increase in thermogenic activity of visceral white adipose tissue (vWAT) but not brown adipose tissue (BAT), at normal room temperature [5]. BAT activity has gained significant attention lately as a general marker of cardiometabolic health, and has been implicated in disorders such as hyperglycemia, hyperlipidemia , heart disease and even certain cancers [6]. Therefore, BAT activity might not be contributing to energy balance post-RYGB under normal daily conditions but might be engaged when temperature changes. To test this hypothesis, we examined the effect of RYGB on BAT sympathetic tone and thermogenic capacity upon cold exposure in diet-induced obese mice. To exclude weight as a confounding variable, we performed our studies in weight-matched sham-operated animals.

## Method

RYGB and sham operations in mice were performed as previously described [1, 2].

### In Vivo Recordings of Brown Adipose Tissue Sympathetic Nerve Activity (BAT SNA) to the Effects of Cooling in the RYGB- and Sham-Operated Mice

Mice anaesthetized with i.p. administration of ketamine (91 mg/kg body weight) and xylazine (9.1 mg/kg body weight) underwent intubation with PE-50 to provide an unimpeded airway for the mouse to spontaneously breathe O_2_-enriched room air. Next, a tapered micro-renathane tubing (MRE-40, Braintree Scientific) was inserted into the right jugular vein for infusion of the sustaining anesthetic agent, α-chloralose (initial dose: 12 mg/kg, then a sustaining dose of 6 mg/kg per h). Another tapered MRE-40 catheter was inserted into the left carotid artery for continuous measurement of arterial pressure and heart rate. Core body temperature was monitored with a rectal probe and continuously maintained at 37.5 °C with a temperature controller (Physitemp Model MCAT2). Each mouse was then equipped for direct multifiber SNA from the nerves serving the sub-scapular BAT. A bipolar platinum-iridium electrode (36-gauge, A-M Systems) was suspended under the nerve and secured with silicone gel (Kwik-Sil, WPI). The electrode was attached to a high-impedance probe (HIP-511, Grass Instruments), and the nerve signal was amplified at 10^5^ times and filtered at 100-Hz and 1,000-Hz cutoffs with a Grass P5 AC pre-amplifier. The amplified and filtered nerve signal was routed to a speaker system and to an oscilloscope (model 54501A, Hewlett-Packard) to monitor the audio and visual quality of the BAT sympathetic nerve recordings for quantification purposes. The nerve signal was also directed to a resetting voltage integrator (University of Iowa Bioengineering, model B600C) to analyze the total activity (integrated voltage) and finally to a MacLab analog-digital converter (ADInstruments, Castle Hill, New South Wales, Australia, Model 8S) containing the software (MacLab Chart Pro; Version 7.0) that utilizes a cursor to count the number of spikes/second that exceeds the background noise threshold. Under a stable plane of anesthesia and strict isothermal conditions (37.5 °C), a continuous recording of baseline BAT SNA was obtained over a 30-min basal period. Each mouse was then gradually cooled (at a rate of −0.25 °C/min) by turning off all external heat sources (surgical heat lamp and heated surgical table) and by placing several chemical ice packs (pre-cooled to −80 °C) in the vicinity of the mouse until the core body temperature reached 27.5 °C. The mouse was then reheated to 37.5 °C with the resumption of all external heat sources and with the removal of all chemical ice packs. At the conclusion of the study, the BAT nerve was cut proximal and distal to the recording site and residual background noise measured to normalize total BAT nerve activity. A group of 8 mice in each arm were performed for this study.

### Radiolabeled 2-Deoxyglucose Uptake Study

Diet-induced obese C57Bl/6J male mice (20–22 weeks old) on high fat diet (Research Diets, 12492) that underwent either RYGB or sham surgery were individually housed in a rodent environmental chamber (Power Scientific) at either 20°C or 30°C for 72 h. Following an overnight fast, tail blood was collected from all mice (time=0). All mice were then injected intraperitoneally with 8–10 μCi of [^3^H]-2-deoxyglucose in a 20% glucose solution, and tail blood was collected over the course of 60 min (15 min, 30 min, and 60 min). At the end of the time course, all mice were sacrificed by decapitation and tissues immediately dissected, flash frozen in liquid nitrogen, and placed at −80 °C until analysis. Plasma radioactivity and tissue-specific uptake of [^3^H]-2-deoxyglucose was measured as previously described [7, 8]. A group of 8 mice in each arm were performed for this study at different temperature settings.

### Western Blot Analysis

Two groups of mice were sacrificed at different temperatures, 1 week after surgery. One group was sacrificed after heating/maintaining a core temperature of 37–38 °C, and the other group was sacrificed after cold exposure with ice bags, until core temperature reached 27–28 °C. Then the corresponding tissues were collected and homogenized in a lysis buffer (50 mM HEPES pH7.5, 150 mM NaCl, 1 mM MgCl2, 1 mM CaCl2, 10 mM NaF, 5 mM EDTA, 1% Triton X-100, 2 mM sodium orthovanadate, and Roche cocktail protease inhibitor tablet). Protein concentrations were measured, and equal amounts were loaded to a 8% SDS PAGE gel for electrophoresis. Proteins on the gel were electro-transferred to a PVDF membrane (Bio-Rad). The blots were then blocked with 5% (w/v) non-fat milk in TBST (0.1% Tween 20) and were probed with anti-TH (2025-THRAB, 1:2,000, PhosphoSolutions, Aurora, CO), β-actin (60008-1-Ig, 1:100,000, Proteintech) or UCP1 (sc-6528, 1:600, Santa Cruz) overnight at 4°C. After washing with TBST, the blots were incubated with a secondary anti-rabbit or anti-mouse IgG antibody (1:10,000) for 2 h. After washing with TBST, protein of interest was imaged with an enhanced chemiluminescence (ECL) detection kit (GE Healthcare, Little Chalfont, UK) on an imager.

### Statistical Analysis

All reported data are presented as mean ± standard error of means (SEM) with statistical analysis by Student’s t test or one-way ANOVA that was followed by the Tukey-Kramer correction analysis if necessary. Figures were generated using GraphPad Prism 9.0 (GraphPad Software), and a *p* value of < 0.05 was considered statistically significant.

## Results

**Fig. 1:**
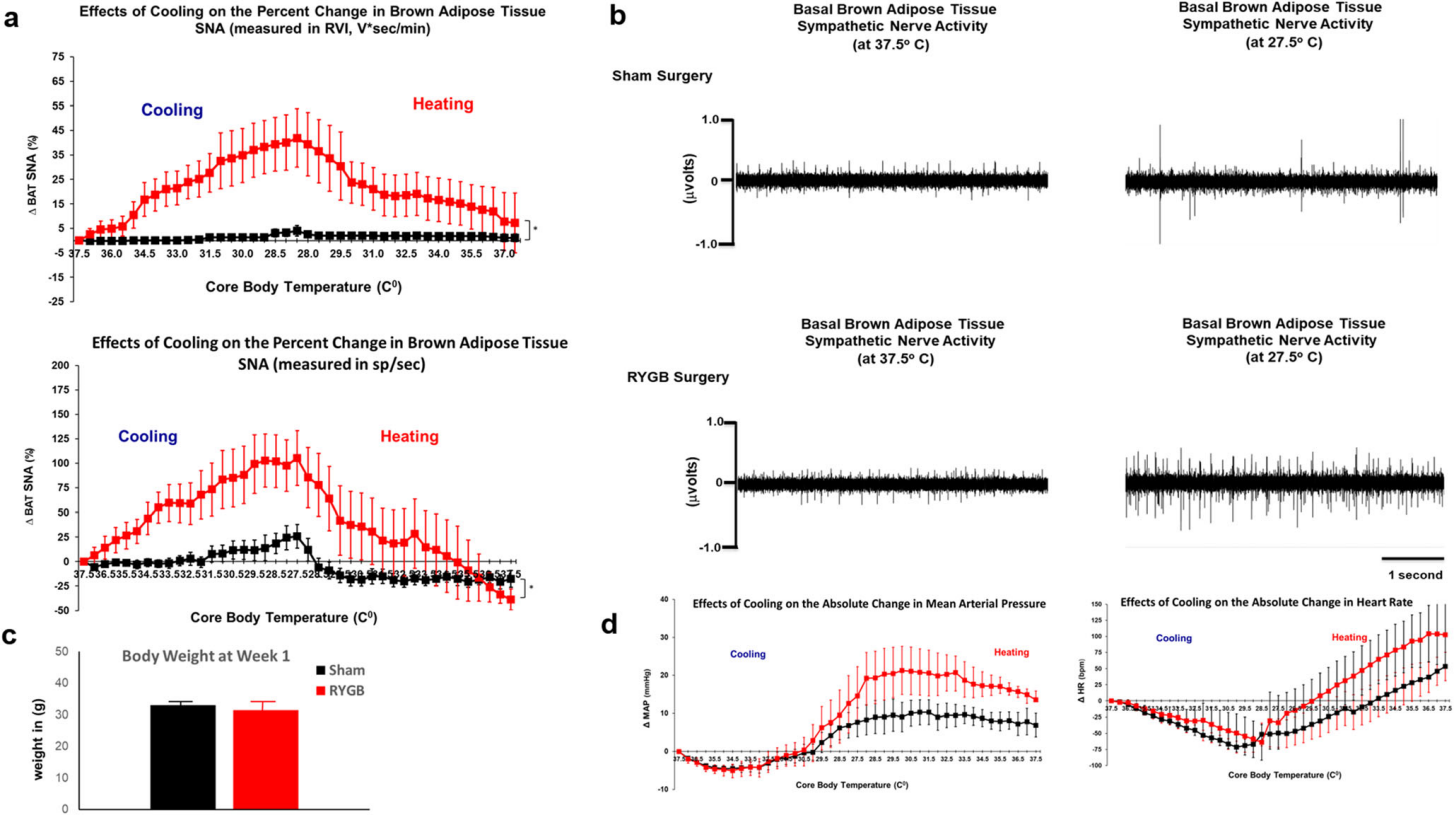
Increased sensitivity of brown adipose tissue (BAT) sympathetic nerve activity (SNA) to cooling after Roux-en-Y gastric bypass. **a** Percent change in SNA of BAT (expressed in RVI, v*s/min (top panel) and in spikes/s (lower panel)) to cooling and heating effect between sham- and RYGB-operated mice, 1 week after surgery. **b** Representative neurogram of BAT sympathetic nerve activity in sham- and RYGB-operated mice at peak hot and peak cool temperature. **c** Body weight of sham- and RYGB-operated mice at week 1 after surgery. **d** Change in mean arterial pressure (MAP) and heart rate (HR) in sham- and RYGB-operated mice during the cooling/heating experiment. One-way ANOVA was used to detect difference between the two groups in **a**. ^*^, *p* <0.05. Sham, n=8; RYGB n=6.

We report that cooling results in a significant increase of BAT sympathetic nerve activity (SNA) in RYGB-operated mice at peak-cold temperature. There was a 40.5% increase in SNA intensity (measured by RVI; V*s/min) and 104% increase in SNA velocity (measured by frequency spikes/s) (*p*=0.0011). On the other hand, re-heating to a normal core body temperature of 37 °C reverses this RYGB-mediated augmentation in SNA (Fig. 1a, b). It is also important to note that this bypass-induced plasticity of sympathetic nerve fibers is independent of surgical-induced weight loss as animals were tested 1-week post-surgery where sham- and RYGB-operated mice were weight-matched (Fig. 1c). Additionally, the cooling did not cause any significant changes in mean arterial blood pressure or heart rate between the two groups (Fig. 1d). This further suggests that this SNA modulation is not due to systemic non-specific activation of the whole autonomic/sympathetic axis.

**Fig. 2:**
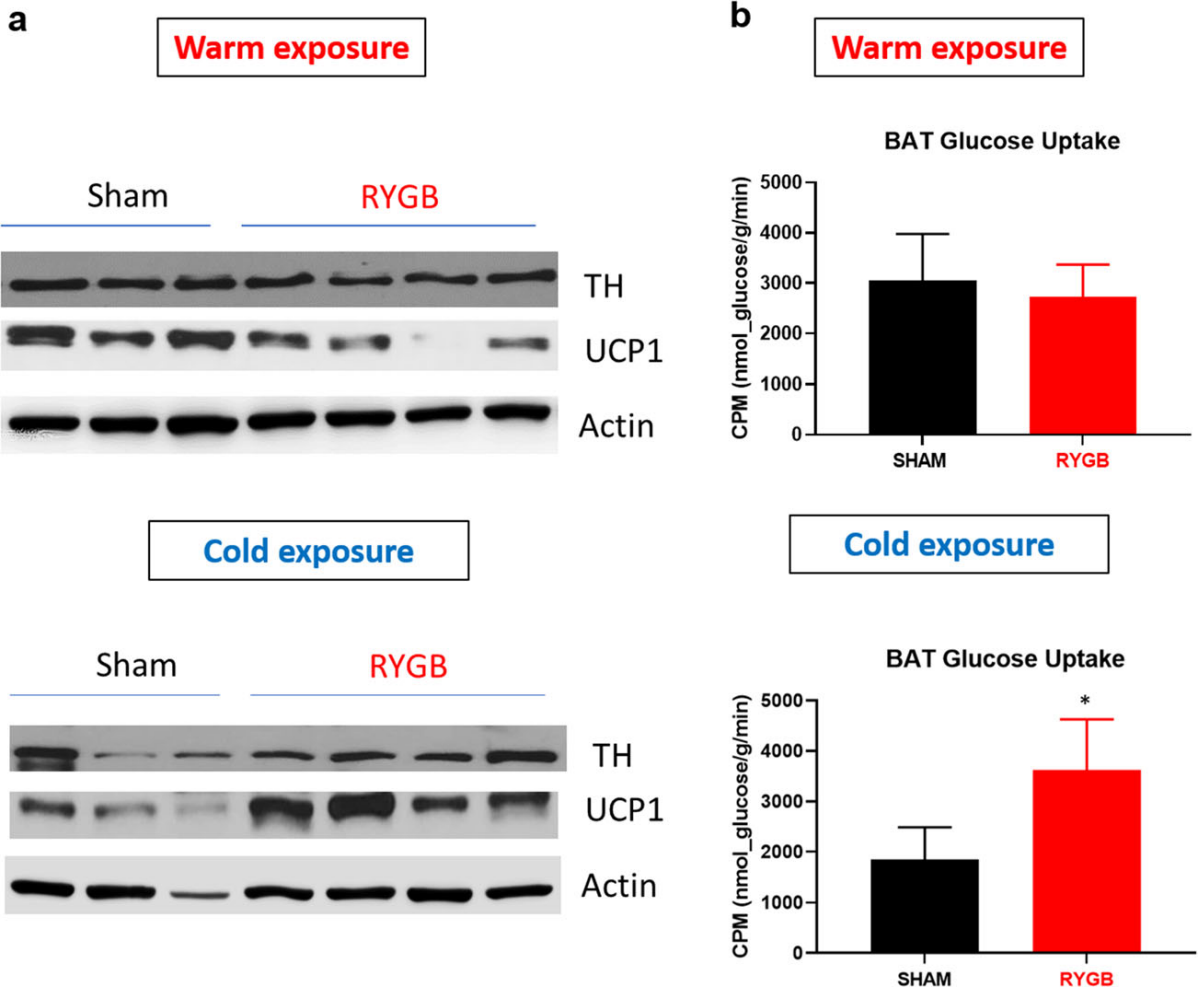
Increase in thermogenic activity of BAT after Roux-en-Y gastric bypass. **a** BAT protein expression of TH and UCP1 by western blot in sham- and RYGB-operated mice sacrificed at core temperature of 37–38 °C (warm exposure) or at 27–28 °C (cold exposure). **b** Radiolabeled [^3^H]-2-deoxyglucose uptake in nmol/g/min by BAT in sham- and RYGB-operated mice 1 week after surgery at 30 °C temperature (warm exposure) and at 20 °C temperature (cold exposure). Student t-test was used to detect difference between the two groups in **b**. ^*^, *p* <0.05. Sham, n=6-8; RYGB n=8.

Moreover, and consistent with the above SNA experiment, cold exposure to a core temperature of 27–28 °C induces thermogenic surge in BAT expression of TH and UCP1 post-RYGB furthermore reinforcing our sympathetic neurogram data above (Fig. 2a). Likewise, 2-deoxyglucose uptake was increased by BAT in RYGB-operated mice compared to their sham counterparts (*p*= 0.038) when experiment was performed under cold but not warm temperature conditions (Fig. 2b).

## Discussion

Here we report that RYGB, one of the most effective and commonly performed bariatric procedures, causes selective activation of autonomic sympathetic activation of brown adipose tissue to induce thermogenesis under cold conditions in a weight-independent manner. We previously showed that browning of white adipose is responsible for baseline increase in thermogenic activity and increased energy expenditure post-RYGB under room temperature conditions [5]. Our recent findings suggest that BAT could also contribute to a further augmentation of energy expenditure induced by RYGB, resulting in its long-term efficacy as a weight loss procedure. Increased activity of BAT has also been tightly associated with improvement of several cardiometabolic outcomes in animals and humans [6, 9]. Enhancing the sensitivity of BAT activity to cold exposure may also contribute to the other cardiometabolic benefits of RYGB.

Being able to understand the underlying regulators of BAT activity in normal physiology (as well as in the setting of bariatric surgery) could help with future development of BAT-targeted therapy that can further improve our current management of obesity and its associated metabolic disorders. In conclusion, our data show that BAT activity is plastic post-RYGB and is independent of surgical-induced weight loss. This bypass-mediated augmentation in BAT activity upon cold exposure might be accountable for the day-to-day variations in thermogenic activity as well as the other metabolic benefits of bariatric surgery.
